# Outbreak of macrolide-resistant mycoplasma pneumoniae in a primary school in Beijing, China in 2018

**DOI:** 10.1186/s12879-019-4473-6

**Published:** 2019-10-22

**Authors:** Wen-Zeng Zhang, Song-Jian Zhang, Quan-Yi Wang, Yin-Dong Li, Hong-Bo Jing, Guang-Yi Hu, Dan Wu

**Affiliations:** 1 Beijing Shunyi Center for Disease Control and Prevention, Beijing, China; 2 Beijing Center for Disease Control and Prevention, Beijing, China

**Keywords:** Mycoplasma pneumonia, Macrolide-resistant, Outbreak investigation, Primary school, China

## Abstract

**Background:**

On 7th June, 2018, a primary school in Beijing, China notified Shunyi CDC of an outbreak of acute respiratory disease characterized by fever and cough among students and resulting in nine hospitalization cases during the preceding 2 weeks. We started an investigation to identify the etiologic agent, find additional cases, develop and implement control measures.

**Methods:**

We defined probable cases as students, teachers and other staffs in the school developed fever (T ≥ 37.5 °C) with cough or sore throat; or a diagnosis of pneumonia during May 1–June 31, 2018. Confirmed cases were probable cases with Mycoplasma pneumoniae detected in oropharyngeal (OP) swabs by quantitative real-time polymerase chain reaction (qPCR). We searched case by reviewing school absenteeism records and interviewing students, teachers and staff in this school. Oropharyngeal swabs were collected from symptomatic students. Two qPCR) assay, a duplex qPCR assay, and sequencing were performed to determine the pathogen, genotype and macrolide resistance at the gene level, respectively.

**Results:**

From May 1st to June 31st, 2018, we identified 55 cases (36 probable and 19 confirmed), of whom 25 (45%) were hospitalized for complications. All cases were students, none of the teachers and other staffs in the school were with similar symptoms. The attack rate (AR) was 3.9% (55/1398) for all students. The cases were mainly male (58%), with an age range of 7–8 years (median: 7 years). 72% (18/25) of inpatients had radiograph findings consistent with pneumonia, and some cases were hospitalized for up to 4 weeks. Pathogen detection results indicated that Mycoplasma pneumonia (M. pneumoniae) P1 type 1 was the causative agent in this outbreak, and the strain harbored one point mutation of A to G at position 2063.

**Conclusions:**

The infections by macrolide-resistant M. pneumoniae are not always mild and pneumonia was common and M. pneumoniae could causes serious complications which require long-term hospitalization. In the future infectious disease prevention and control practice, M. pneumoniae should be paid more attention. It is necessary to establish and improve the pathogen and drug resistance surveillance system in order to prevent and control such mutated strains of M. pneumoniae from causing future outbreaks or epidemics in China.

## Background

Mycoplasma pneumoniae (M. pneumoniae) is a common pathogen of human respiratory tract infections (RTIs) and is a leading cause of community-acquired pneumonia (CAP) [1–3]. M. pneumoniae infections develop in persons of all ages, especially in children and teenagers [4]. M. pneumoniae infections range clinically from mild, self-limiting respiratory symptoms to radiographically confirmed pneumonia in an estimated 30% of cases [5]. In rare cases, M. pneumoniae can cause extrapulmonary manifestations, including neurologic, dermatologic, hematologic and cardiac syndromes which can result in hospitalization and death [6, 7]. Macrolide antibiotics are commonly used drugs for the treatment of M. pneumoniae infection. With the widespread or inappropriate use of antibiotic, macrolide-resistant M. pneumoniae (MRMP) has become an emerging threat worldwide [8–10]. In recent years, MRMP has become very serious in Asia [11–14].

M. pneumoniae is commonly associated with acute respiratory disease (ARD) and pneumonia outbreaks in semi-closed and closed settings such as care facility, kindergartens, schools and universities [10, 15, 16]. MRMP strain has been identified in some pneumonia outbreaks in recent years [10, 17]. In China, M. pneumoniae infection is not a notifiable disease, most patients infected with MP are seldom symptomatic, patients rarely seek medical attention, and the clinical syndrome overlaps with other pneumonia etiologies, so the M. pneumoniae infection prevalence in the community was widely underestimated. However, several recent outbreaks of M. pneumoniae infections among children and adults have been reported, and macrolide-resistant strains have emerged [18, 19].

On 7 June, 2018, a primary school in Beijing, China notified Shunyi center for disease control and prevention (CDC) of an outbreak of acute respiratory disease characterized by fever and cough among students and resulting in nine hospitalization cases during the preceding 2 weeks. As part of the outbreak response, we conducted an epidemiologic investigation to identify the etiologic agent, find additional cases, develop and implement control measures. This is the largest outbreak of a macrolide-resistant strain of M. pneumoniae at a primary school in China in recent years.

## Methods

### Epidemiologic investigation

#### General situation

The school has a total of 33 classes, including grades 1–5.All students are day students. There are two teaching buildings in the school. The first and second grades are located in the A teaching building, and the remaining grades are located in the B teaching building. At the time of the outbreak, the primary school had 1398 students and 100 teachers and other staffs. A school doctor provides students with medical care.

#### Outbreak finding/outbreak confirmation

On 7 June, we identified 21 students suffering from a respiratory disease characterized by fever and cough in the Class 8 and 9 of Grade 1. Oropharyngeal (throat) swabs were collected from consenting patients presenting with ARD and specimens were tested using two multiplex quantitative polymerase chain reaction (qPCR) assay for 24 common respiratory pathogens (including 9 viruses, 14 bacteria and Pneumocystis pneumonia) at Shunyi CDC to identify the causative agent. Nine of the 12 (75%) specimens were positive for M. pneumonia. We confirmed a M. pneumoniae outbreak in that school according to epidemiological and laboratory data.

#### Case definition and finding

To investigate the outbreak, the school absenteeism records were reviewed, students, teachers and other staffs in the school were interviewed, beginning June 8, 2018, to find cases of acute respiratory disease among students. The outpatient and inpatient records were reviewed and doctors in the hospitals were interviewed. The information regarding demographics, the process of illness, signs and symptoms, underlying conditions, clinical treatments, and extracurricular custody was collected. Retrospective record review was performed to identify cases diagnosed as early as May 1, 2018.

We defined probable cases as students, teachers and other staffs in the school developed fever (T ≥ 37.5 °C) with cough or sore throat; or a diagnosis of pneumonia during May 1–June 31, 2018. Confirmed cases were probable cases with M. pneumoniae detected in oropharyngeal swabs by qPCR.

#### Specimen collection and nucleic acid extraction

Oropharyngeal (OP) swabs were obtained from the students identified as probable cases and their close contacts who agreed to testing. OP swabs were placed in 2 ml of universal transport medium (UTM) and transported at 4 °C to the laboratory of the Shunyi CDC. Total nucleic acid (TNA) was extracted from 200 μl of UTM from each swab specimen and eluted into 100 μl using a MagNA Pure compact total nucleic acid isolation kit (Roche Applied Science) in accordance with the manufacturer instructions.

#### Detection and genotyping

All patient specimens were screened using two multiplex combined qPCR detection kits for Respiratory viruses and bacteria respectively (number product code: CN12–33 and CN13–3, Uninovo Biological Technology Co. Ltd., Jiangsu, China) in order to identify the etiology of the outbreak. The kit for respiratory bacteria were specific for 15 respiratory pathogens, including: *acinetobacter baumannii (Ab)*, *chlamydia pneumoniae(C. pneumoniae)*, *escherichia coli *(*E. coli*), *haemophilus influenza (Hi)*, *Klebsiella pneumoniae* *(KP)*, *Legionella pneumophila* *(Lp)*, *moraxella catarrhalis (MC)*,* mycoplasma pneumoniae* *(M. pneumoniae)*, *Mycobacterium tuberculosis (Mtb)*, *Staphylococcus aureus (S.aureus)*, *streptococcus pneumoniae* (*S. pneumoniae*), *Streptococcus pyogenes* *(SP)*, *stenotrophomonas maltophilia (SMA)*, *pseudomonas aeruginosa* *(PAE)*, and *pneumocystis jiroveci pneumonia (PCP)*. The kit for respiratory viruses were specific for 9 respiratory viruses, including: *human parainfluenza virus types 1 to 4* *(HPIV)*, *adenovirus (ADV)*, *metapneumovirus (MPV)*, *human coronavirus types OC43/NL63/229E/HKU1* *(HCOV)*, *respiratory syncytial virus (RSV)*,* human rhinovirus* *(HRV)*, *influenza virus types A/B/C* *(FLU)*, *human enterovirus* *(HEV)*, and *human bocavirus (HBOV)*. The multiplex qPCR was performed by previously described methods [20]. A duplex qPCR assay was used for P1 genotyping of M. pneumonia, and primers and probes were as previously described [21].

#### Detection of macrolide resistance at the gene level

Point mutations associated with resistance in domain V of 23S rRNA were detected. Genomic DNA of all M. pneumoniae was extracted using a QIAamp DNA minikit (Qiagen, Hilden Germany). The extracts were distributed into aliquots and saved at-20 °C. A forward primer extending from position 1758 to position 1775 (GCAGTGAAGAACGAGGGG) and a verse primer extending from position 2684 to position 2664 (GTCCTCGCTTCGGTCCTCTCG) in the sequence of M. pneumoniae 23S rRNA were used to amplify the domain V region of the 23S rRNA gene by PCR methods, as previously described [22]. The amplification products were sequenced by the Invitrogen Life Technologies (Shanghai, China).

### Statistical analysis

All data were analyzed with SPSS 25.0 software (IBM, USA). Categorical variables were described with counts and percentages. Differences between medians were tested by the Mann-Whitney U test, and the Chi-square or Fisher’s exact test was used to compare categorical variables. A *P* value of less than 0.05 was considered statistically significant.

## Results

### Epidemiologic investigation

**Fig. 1:**
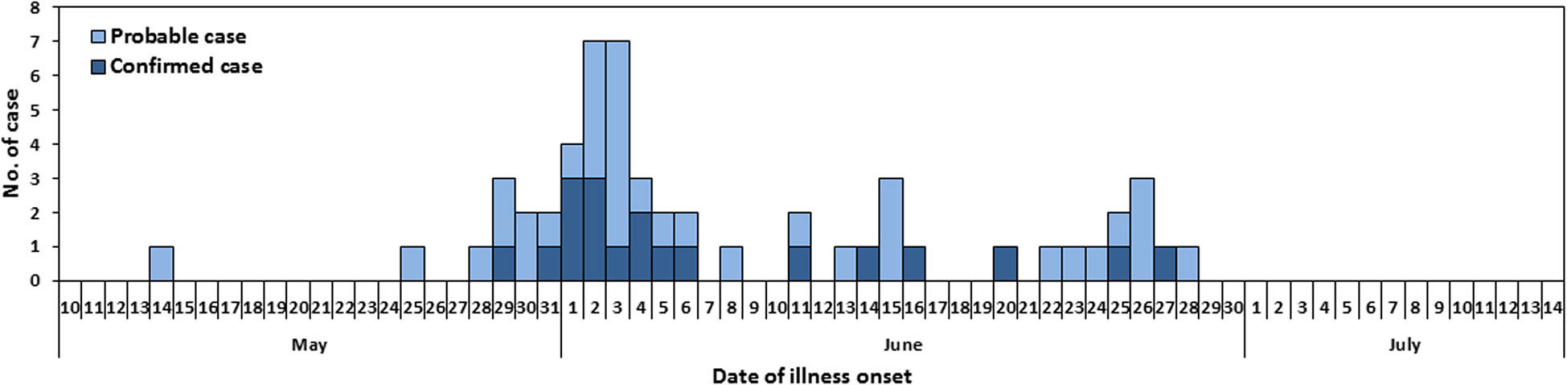
Timeline of Mycoplasma pneumoniae outbreak in a primary school—Beijing, May–June 2018

During May 1–June 31, 2018, a total of 55 cases were identified, including 19 confirmed and 36 probable cases. Illness onset occurred during May 14–June 28, 2018 and peaked at the beginning of June (Fig. 1). The first child developed ARD on 14 May, 2018, rested at home on the 15th, and returned to school on the 16th. This child is a student of class 8 grade 1, and a member of sunflower dinner table. The “sunflower dinner table” here is a private institution which makes profit by providing lunch and dinner to students in noon and evening after school time, and it also provides space for students to have a nap in the noon. This patient, had a high fever (39.0 °C), sore throat, and cough, went to Shunyi maternal and child health Hospital of Beijing Children’s Hospital, Beijing, China, for outpatient treatment on the afternoon of May 16, 2018. The initial diagnosis was bronchitis, and the later diagnosis was mycoplasma pneumonia. On May 28, the child’s symptoms improved and returned to the school. With the resumption of the first patient, 54 cases with symptoms of ARD and pneumonia occurred one after another.

All 55 probable and confirmed cases of M. pneumoniae were students, none of the teachers and other staffs in the school exhibited similar symptoms. The attack rate (AR) of all students was 3.9%(55/1398)**.** Of the 55 cases, 48 (87%) were grade 1 students, 7 (13%) were grade 2 students. The outbreak involved 4 classes in the grade 1 and 2. The overall attack rate of class was 22% (55/248) (range = 2–58%). Among the 55 cases, 10(18%) were members of the sunflower dinner table. The AR of the sunflower table students was 23% (8/35). There was no significant difference in the attack rate between the two groups (Chi-Squares = 0.01,*P* = 0.928). The cases were mainly male (58%), with an age range of 7–8 years (median: 7 years), the above distribution characteristics are basically consistent with the distribution characteristics of all students.

A total of 53 patients (96%) reported cough, 50 (91%) fever, and 24 (44%) sore throat. Twenty-five (45%) of the patients were hospitalized for complications, including 2 with abnormal myocardial biomarker (CK-MB: > 30 U/L) and 1 of whom with pleural effusion; eventually all cases were recovered. Radiographs were administered for all 25 inpatients with pneumonia diagnoses; 18 (72%) of whom had findings consistent with pneumonia with radiographical indications. Eighteen (72%) inpatients were treated with azithromycin, 9 (36%) with beta-lactams, 9 (36%) with multiple antibiotics, and 13 (52%) with glucocorticoid, none of the patients was treated with quinolones or tetracyclines but the symptoms such as cough lasted for a long time in most cases, and some cases were hospitalized for up to 4 weeks (Table 1).

**Table 1:** Number and percentage of patients with confirmed or probable M. pneumoniae cases among students in a primary school, by selected characteristics — Beijing, 2018

Characteristic	Confirmed (*n* = 19)		Probable (*n* = 36)		Total (*N* = 55)	
	No.	(%)	No.	(%)	No.	(%)
Sex						
Men	12	(63)	21	(58)	33	(60)
Women	7	(37)	15)	(42	22	(40)
Grade						
Grade 1	17	(89)	31	(86)	48	(87)
Grade 2	2	(11)	5	(14)	7	(13)
Symptom						
Cough	18	(95)	35	(97)	53	(96)
Fever	17	(89)	33	(92)	50	(91)
Sore throat	10	(53)	14	(39)	24	(44)
Pharyngeal wall congestion	7	(37)	8	(22)	15)	(27
Body aches	6	(32)	7	(19)	13	(24)
Headache	6	(32)	6	(17)	12	(22)
Tonsil enlargement	6	(32)	6	(17)	12	(22)
Runny nose	2	(11)	2	(6)	4	(7)
Nasal congestion	0	(0)	2	(6)	2	(4)
Chest radiograph						
No. of patients administered	10	(53)	15	(42)	25	(45)
No. with findings consistent with pneumonia	7	(70)	11	(73)	18	(72)
Outcome						
Outpatient	9	(47)	21	(58)	30	(55)
Hospitalization	10	(53)	15	(42)	25	(45)
Antibiotic treatment^a^						
Azithromycin	8	(80)	10	(67)	18	(72)
Beta-lactams^b^	4	(40)	5	(33)	9	(36)
Multiple antibiotics	4	(40)	5	(33)	9	(36)
Glucocorticoid^c^	5	(50)	8	(53)	13	(52)
Information missing	1	(10)	5	(33)	6	(24)

^a^Data from 25 hospitalized cases and the list of antibiotics is not mutually exclusive

^b^Included latamoxef sodium, ceftizoxime, and cefmetazole

^c^Included prednisone and methylprednisolone

The median hospitalization period was 11 days (range = 2–28 days), and the median duration of disease was 16 days (range = 6–34 days).Among the 55 cases, the overall median time interval between onset to first visit to any medical institution was 1 day (range = 0-10 days), the time interval before June 8 was longer than cases onset on and after June 8 (Z = -3.238,*P* < 0.001).

Oropharyngeal swabs were collected from 56% (31/55) of all cases and tested by qPCR, 19 (61%) specimens were positive for M. pneumoniae and no other pathogens were identified. Nineteen positive specimens included 14 cases suffering from pneumonia (14/25), 5 with URI (5/30). One (5%) of 20 close contact specimens was positive for M. pneumonia and the student was finally judged to be a carrier. The positive rate of mycoplasma in pneumonia cases was significantly higher than that of patients with URI and carriers (chi-square = 6.36 and chi-square = 24.18; *P* < 0.01 and *P* < 0.001). All 19 strains were classified as P1 type 1 by P1 genotyping of the primary specimens. The result of nucleotide sequence analysis of the domain V region of the 23S rRNA gene in 7 primary specimens showed that the strain harbored one point mutation of A to G at position 2063.

### Public health response

On June 8, 2018, Shunyi CDC guided the school to take the following prevention and control measures, including closure of two classes with the most morbidity in the first grade for 2 weeks, suspending of group activities, reinforcement of ventilation and disinfection in all classrooms and education regarding good health behaviors to reduce the risk of disease transmission. All cases were promptly treated outpatient or inpatient and discharged from school until afebrile for ≥48 h. Shunyi CDC communicated with parents of students about respiratory infection prevention and control measures, including good hand hygiene and respiratory hygiene, and seeking medical attention when you have a cough and fever. Six dinner tables with cases were closed. Shunyi CDC and the relevant departments informed the dinner table around the school the epidemic situation. In order to characterize the possible continuing circulation of M. pneumoniae and identify the potential need for adjustments to control measures, an active surveillance for pneumonia was established in the primary school. The measure of screening the students for fever and active respiratory symptoms when students arrived at school was implemented.

## Discussion

In China, M. pneumoniae infection is not a notifiable disease, so the prevalence of M. pneumoniae infection in schools is unclear and the severity of M. pneumoniae outbreaks was still under discussion. However, several outbreaks of M. pneumoniae have been reported at schools in China, and macrolide-resistant strains have emerged [18, 19]. This is the largest outbreak of a macrolide-resistant strain of M. pneumoniae at a primary school in China in recent years and this outbreak attracted widespread concerns from local governments, health administrative departments and public health departments. After taking control measures, the number of cases decreased gradually, and the time interval between onset to first visit to any medical institution was significantly shortened.

M. pneumoniae is spread through respiratory droplets; therefore, physical barriers may be effective for blocking spread of infection, which would be helpful in management of M. pneumoniae outbreak. Wang et al. [18] found that the major risk factor was the distance (less than 3 m) between the school children at an outbreak of M. pneumoniae in a nursery school. In our study, the field epidemiological investigation result showed that the outbreak began in class 8 grade 1 and the sunflower dinner table where the first case studied and rest. Subsequently, cases began to appear in the neighboring class. This result was different from previous research result of outbreaks in two primary schools [19]. This result indicates that good environmental and hygienic conditions are important for the prevention and control of respiratory infections disease outbreaks.

This M. pneumoniae outbreak was identified after a significant number of acute respiratory disease cases were reported among students at Shunyi CDC. The multiplex qPCR assay for respiratory viruses and bacteria was used as the primary testing method in this investigation. The multiplex qPCR assay was useful for outbreaks in which the etiology is initially unknown, like this one, because it enables simultaneous testing for multiple pathogens within a specimen. After the outbreak’s etiology was confirmed, active surveillance for probable cases and specimen collection for diagnostic testing began and clinicians frequently prescribed antibiotics on the basis of reports of a fever or cough rather than on patient evaluation or diagnostic test results. In our study, the positive rate of M. pneumoniae in pneumonia cases was significantly higher than that of students with URI and carriers and all 19 strains were classified as P1 type 1. A reduction in the time between outbreak recognition and initiation of active surveillance with prioritized specimen collection will lead to appropriate patient treatment and implementation of timely and strict infection control measures to prevent morbidity and mortality.

MRMP isolates were first found in 1968 and various rates of macrolide resistance have been reported in recent M. pneumoniae surveillance [22]. Walter et al. reported that erythromycin-resistant strains of M. pneumoniae increased from 0% in 2002 to 30.6% in 2006 in Japan [23]. Zheng et al. reported that the MRMP rates increased from 17% in 2005 to 76% in 2006 and 100% in 2007 and 2008 in Shanghai, China [11].Wang et al. reported that the MRMP was at a high rate of more than 90% from 2008 to 2012 in Beijing [19]. The above research results showed that MRMP spread rapidly in parts of Asia. However, there have been few reports of outbreaks caused by MRMP in China. The strain isolated from this outbreak was macrolide resistant. In this outbreak, the more severe clinical signs and symptoms, the more hospitalizations with complications, the longer period of hospitalization, the longer duration of disease of all patients, and these results may be related to above finding.

Some studies have shown that the symptoms of infected macrolide-resistant strains are more severe than those of macrolide-sensitive strains [24–26]. In our study, pneumonia was most common symptoms among students who tested positive for M. pneumoniae strains during the outbreak. One of the cases was diagnosed as RMPP and hospitalized for 28 days for myocardial damage and pleural effusion. The results indicated that the clinical manifestations of patients infected with M. pneumoniae are not always mild, and M. pneumoniae could causes serious complications requiring long-term hospitalization. In the future epidemic prevention and control work, M. pneumoniae should be paid more attention. It is necessary to establish and improve the test, report, and surveillance system for M. pneumoniae in China.

There were still a few limitations in our study. First and foremost, we did not collect specimens from every on of all 55 cases, only 56% cases had specimens tested by qPCR. Secondly, the M. pneumoniae isolation and culture work was not conducted, and the drug resistance test based on the in vitro drug sensitivity test was not carried out. All drug resistance results were based on nucleotide sequencing results and previous literature reports. Thirdly, some cases might have been infected in the community, but this probability is low because of the epidemiological time and person distribution and genotype identification.

## Conclusions

This was a macrolide-resistant Mycoplasma pneumoniae outbreak that occurred in a primary school. The infections by macrolide-resistant strains are not always mild and pneumonia was common. M. pneumoniae could cause serious complications which require long-term hospitalization. We recommended Ministry of Health to establish and improve the pathogen and drug resistance surveillance system in order to prevent and control such mutated strains of M. pneumoniae from causing future outbreaks or epidemics in China.

## Data Availability

The datasets used and analyzed during the current study are available from the corresponding author upon reasonable request.

## Abbreviations

AR: Attack rate
CAP: Community-acquired pneumonia
CDC: Centers for Diseases Control and Prevention
CK-MB: Creatine kinase-MB
M. pneumonia: Mycoplasma pneumonia
MRMP: Macrolide-resistant M. pneumonia
OP: Oropharyngeal
RMPP: Refractory Mycoplasma pneumoniae pneumonia
RTIs: Respiratory tract infections
URI: Upper respiratory tract infection
